# Survival of *Trypanosoma cruzi* in Dead *Triatoma gerstaeckeri* Under Laboratory Conditions

**DOI:** 10.4269/ajtmh.25-0370

**Published:** 2025-10-28

**Authors:** Keswick C. Killets, Kaitlyn M. Perez-Rascón, Jose G. Juarez, Gabriel L. Hamer, Sarah A. Hamer

**Affiliations:** ^1^ Department of Veterinary Integrative Biosciences, Texas A&M University, College Station, Texas;; ^2^ Department of Entomology, Texas A&M University, College Station, Texas

## Abstract

*Trypanosoma cruzi* (*T. cruzi*) is primarily transmitted through infectious triatomine feces, with ingestion of infected triatomines being an alternative transmission route. Viable *T. cruzi* inside dead triatomines presents a potential veterinary health concern to animals that may eat insects, especially if insecticides create an abundance of dead insects in the immediate proximity of a host species. In the present study, *Triatoma gerstaeckeri* were experimentally infected with *T. cruzi* and then decapitated; subsequently, their carcasses were subjected to ambient laboratory conditions for intervals of up to 14 days, with feces and gut contents inoculated into culture. Viable parasites were detected throughout a period of 10 days but not thereafter, and the proportion of culture-positive dead triatomines decreased after 6 days (*P* <0.001) compared to 0 days. The odds of parasite viability decreased with the presence of bacterial or fungal growth (*P* <0.001) and at higher cycle threshold values of fecal spots (*P* = 0.008). Dead triatomines may pose a *T. cruzi* transmission risk if ingested by animals.

## INTRODUCTION

*Trypanosoma cruzi *(*T. cruzi*), a protozoan parasite belonging to the family Trypanosomatidae, is the etiologic agent of Chagas disease.^1^ This is a human neglected tropical disease that also affects dogs and other mammals.^2^ The primary mode of transmission occurs via the stercorarian route and is facilitated by triatomine bugs (Hemiptera: Reduviidae). In this route, *T. cruzi* in feces is introduced to hosts through a bite wound, mucous membrane, or the oral ingestion of infected material.^3^^,^^4^ The survival of *T. cruzi* after triatomines have died is of potential veterinary and public health interest. In some studies, *T. cruzi* was shown to live in the intestines of dead triatomines for up to 9 days,^5^^–^^7^ or even up to 30 days in the fifth instar of *Triatoma infestans *(*T. infestans*).^8^ Recently, cultured viable *T. cruzi* from a dead *Paratriatoma lecticularia* was found in a dog kennel in South Texas.^9^ This result reveals that dead triatomines with viable parasites are in immediate proximity to susceptible hosts, leading to a potential risk of transmission. In the current study, the long-term survival of *T. cruzi* in deceased *Triatoma gerstaeckeri *(*T. gerstaeckeri*) is explored under laboratory conditions. This species is epidemiologically important in parts of the southern United States^3^ and Mexico,^10^ contributing to sylvatic and peridomestic transmission cycles.

Fifth instar nymphs of *T. gerstaeckeri *that had been reared in an arthropod containment level 2 facility since 2020 and were at least F1 generations were used in the present study. Triatomines were reared under a 12-hour light cycle, within a temperature range of 25–27°C, and maintenance blood feeding was accomplished using an artificial membrane feeder with defibrinated rabbit blood. Triatomines were experimentally infected with rabbit blood spiked with epimastigotes of *T. cruzi* discrete typing unit TcI, which was acquired from a triatomine collected in Texas,^11^ via an artificial membrane feeder according to previously published protocols.^12^ To confirm that each triatomine acquired the infection, either a voided fecal spot on filter paper or feces obtained via abdominal compression was collected and tested using polymerase chain reaction (PCR) testing, with an average time interval between experimental infection and feces collection of 14.7 days to allow for colonization of the infection. Feces were subjected to DNA extraction and tested via quantitative PCR (qPCR) to detect *T. cruzi*.^11^^,^^13^ Only triatomines with positive results were included in the current study. If a triatomine’s feces initially tested negative for *T. cruzi*, a second feeding with *T. cruzi*-spiked blood was provided, and the same molecular confirmatory procedures were performed.

The present study involved a total of 141 triatomines tested in 14 independent trials (4–20 triatomines per trial) because of the staggered availability of *T. cruzi*-confirmed *T. gerstaeckeri*. Each trial involved the same procedure for specimen killing, assignment to one of 10 time points (0, 1, 2, 3, 4, 5, 6, 7, 10, or 14 days), temperature and humidity recording, engorgement evaluation, *T. cruzi* culture, and quality assurance. Triatomines were assigned an engorgement score (1: none, 2: a little engorged, 3: medium engorged, 4: fully engorged) on the basis of the degree of abdominal distension. All trials included at least one insect assigned to the 0-hour time point. Because positive cultures were consistently detected at earlier time points, longer time points were incorporated progressively, extending the assessment period up to 14 days.

At the beginning of each trial, triatomines were killed in a biosafety cabinet, and all tools were flame-sterilized before use. Triatomines were severed distally to the forelegs (later referred to as “decapitation”) and placed in individual petri dishes, with lids off, which marked the start of the trial. All triatomines except those assigned to the 0-hour time point were moved into an enclosed, clear plastic PCR cabinet inside the laboratory under ambient conditions. The temperature and relative humidity were recorded when the triatomines were placed in and taken out of the cabinet, and these latter values were used in data analysis.

Once the assigned time point was reached, triatomine carcasses were retrieved, and two different biological samples from each triatomine were inoculated into culture media: feces and guts. First, fecal material was expelled from the triatomine’s body by compression for inoculation into a 5 mL flask of liver infusion tryptose (LIT) media^14^ supplemented with fetal bovine serum, which was treated with three times the normal concentration of penicillin–streptomycin (60 *µ*L) and nystatin (1.5 mL) per 1 L of LIT media (Sigma-Aldrich, Darmstadt, Germany).^9^ In cases in which no fecal material could be expelled, the distal segment of the abdominal exoskeleton of the triatomine was placed into the media. Second, gut contents were retrieved from the triatomine’s abdomen by dissecting it with a new set of sterile tools and then placed into a new flask of LIT media. Cultures were incubated at 27°C^15^ and examined weekly for up to 2 months via microscopy to detect the presence of viable parasites, which consisted of motile life stages, primarily epimastigotes.

Data were initially evaluated using descriptive statistics to assess frequency distributions and facilitate visualization. *Trypanosoma cruzi* viability was evaluated using logistic regression analysis with a binomial distribution, reported as odds ratios (OR) and at 95% confidence intervals (CIs). Fixed effects included the time point, presence of other growth (bacterial or fungal), and engorgement score, with covariates including the qPCR cycle threshold (Ct) value of the fecal sample, humidity, and temperature. Model simplification was performed using backward elimination,^16^ in which nonsignificant parameters were removed on the basis of the significance of fixed effects estimates when α = 0.05. Variables were evaluated for collinearity, and the best fit model was selected on the basis of the lowest Akaike information criterion (AIC), a metric for model selection that balances goodness-of-fit with the number of parameters.^17^ All models were generated using R 4.4.3 (R Foundation, Vienna, Austria).^18^

**Figure 1.**
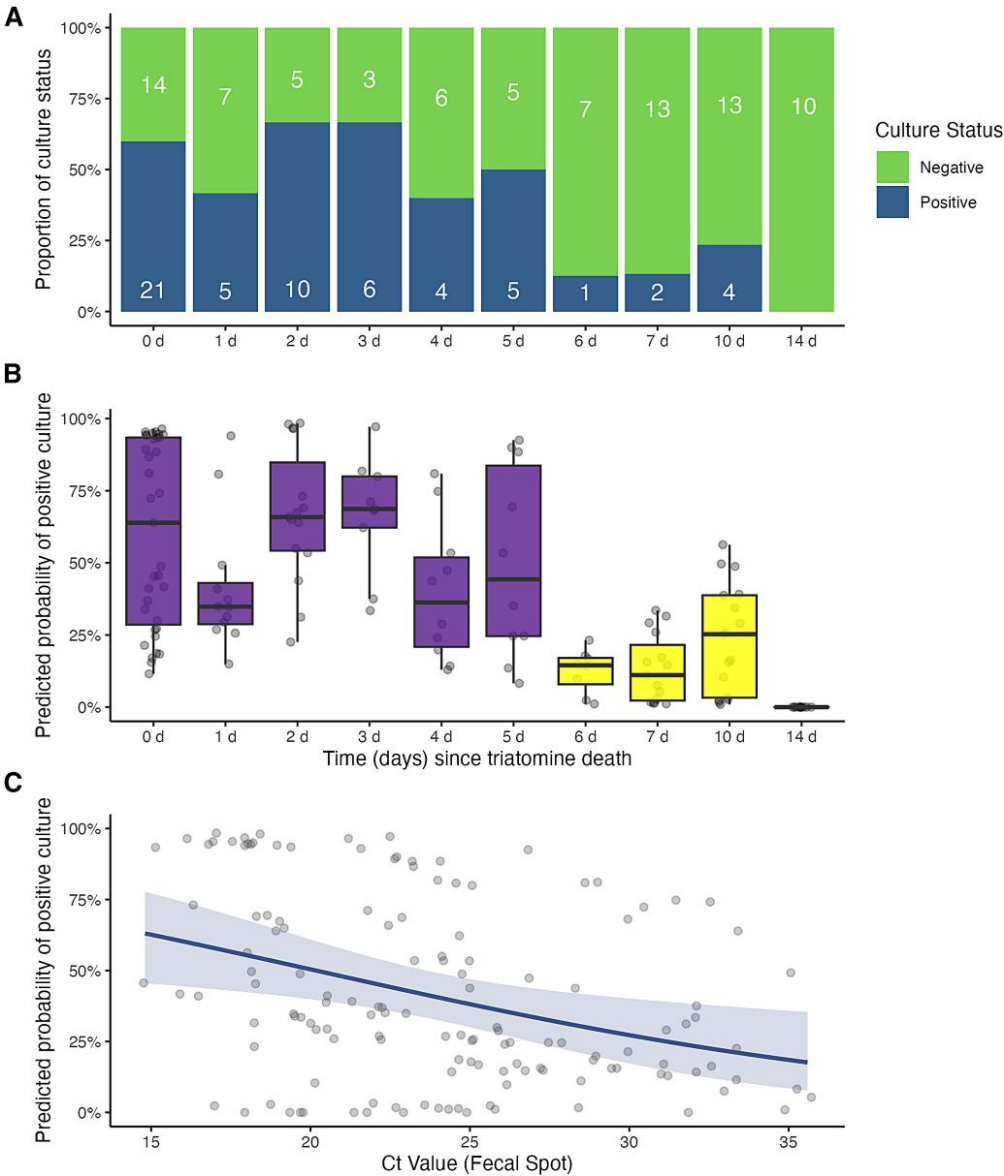
(**A**) Culture status proportions (for the viability of *Trypanosoma cruzi *[*T. cruzi*]) for each of the time points (days) since the death of the infected *Triatoma gerstaeckeri* (*T. gerstaeckeri*). If either the fecal or gut culture was positive for a triatomine, the overall status was considered positive. (**B**) Boxplots of the predicted probability of having a *T. cruzi*-positive culture for each of the time points (days) since the death of the infected *T. gerstaeckeri*. The statistical significance for *T. cruzi*-positive cultures is denoted in purple (no statistical significance) and yellow (statistical significance). (**C**) Scatterplot of the predicted probability of having a *T. cruzi*-positive culture based on the cycle threshold value of the quantitative polymerase chain reaction-positive feces that was tested before the trials.

A total of 141 triatomines were used for the trials, of which 58 (41.1%) across all time points had viable *T. cruzi* based on either feces or gut culture inoculations (Figure 1A). A total of 42 fecal cultures and 46 gut cultures were analyzed, revealing viable *T. cruzi* in 29 triatomines (20.6%) that had positive fecal and gut cultures. Cultures performed on 75 triatomines revealed some form of bacterial or fungal growth (fecal or gut); 24 of these triatomines (32%) had viable *T. cruzi* in their cultures.

The best-fit model for the effects impacting *T. cruzi* survival included the time point, overall bacterial or fungal growth, and Ct value of the tested feces sample as significant explanatory variables. At the 6-day (OR = 0.03; CI = 0.001–0.27; *P* = 0.006), 7-day (OR = 0.03; CI = 0.003–0.21; *P* <0.001), and 10-day (OR = 0.08; CI = 0.13–0.39; *P* = 0.004) time points, the odds of parasite viability were >92% lower than in cultures at earlier time points, respectively (Figure 1B). The odds of parasite viability in cultures with bacterial or fungal growth were 93% lower than in cultures without microbe growth (OR = 0.07; CI = 0.02–0.22; *P* <0.001). Finally, for every one-unit increase in the qPCR Ct value of the feces samples tested before the trials, the odds of parasite viability decreased by 12% in the cultures (Figure 1C; OR = 0.88; CI = 0.79–0.96; *P* = 0.008).

With more than 100 years since its discovery and classification,^19^ Chagas disease and its causative agent, *T. cruzi*, remain a serious public and veterinary health threat, with many mechanisms of transmission poorly understood. The present study revealed that *T. cruzi* can remain viable in dead *T. gerstaeckeri *for up to 10 days after the death of the triatomine, a finding previously observed in other triatomine species.^5^^,^^7^ The probability of positive *T. cruzi* culture also significantly decreased after 6 days. Although parasite viability was not observed beyond 10 days in the study cultures, parasites could live for up to 30 days in dead *T. infestans *that had been euthanized using insecticide.^8^ This study’s specimen killing method was decapitation, which left an opening vulnerable to exposure to environmental effects^5^ and possibly influenced *T. cruzi* survivorship given increased desiccation. It is important to note that this study’s findings were generated under stable laboratory conditions and may not fully reflect the environmental variability encountered by triatomines in the field. In particular, high ambient temperatures and low humidity, often found in Texas, are likely to reduce parasite survival post-mortem. Future work can be conducted to investigate how variable storage conditions influence *T. cruzi* survivorship, especially after triatomine mortality due to insecticides. Regardless, these findings reveal that aged, dead infected triatomines may pose a risk for *T. cruzi*. These results highlight the need to use personal protective equipment (e.g., gloves) when handling dead triatomines and raise concerns regarding potential transmission if infected dead triatomines are consumed. This transmission risk has previously been modeled in the context of using systemic insecticides on dogs to reduce vector populations, with one study revealing increased incidence of infections in dogs if they consumed 10% or more of dead triatomines^20^ and another revealing that longer life spans in dogs increase the chance of infections resulting from the consumption of infected dead triatomines.^21^ Additionally, wildlife can consume triatomines and potentially become infected, thus contributing to the *T. cruzi *sylvatic transmission cycle.^22^

Viable parasites were not detected in every triatomine in the 0-hour time point group, despite immediate inoculations upon decapitation. Similar to observations in a recent study, a high frequency of cultures with bacterial or fungal growth was observed in the present study, even when the LIT media was treated with higher dosages of antibiotics and antifungals.^9^ This may have reduced the number of cultures in which viable *T. cruzi* could be detected, as the parasite may have been outcompeted by the growth of other organisms. Another possibility is that higher dosages of antimicrobials hindered *T. cruzi* growth, as nystatin could possibly be an effective trypanocidal drug.^23^ Furthermore, some triatomines may not have had an established *T. cruzi* infection, despite having a qPCR-positive fecal spot after an average interval of 2 weeks between the infected bloodmeal and decapitation. Despite the limitations and challenges in demonstrating the viability of the parasite, more than 40% of triatomines had positive cultures. Future studies could benefit from the incorporation of alternative culture techniques that minimize contamination without antimycotics, such as motility-based separation in U- or V-shaped tubes, filtration through 3 *µ*m pore membranes, or density gradient purification using Percoll or iodixanol. Moreover, RNA-based viability assays (e.g., reverse transcriptase quantitative PCR targeting labile transcripts) could provide a sensitive and cultivation-independent measure of parasite viability in dead vectors. Incorporating such methods would help validate and expand upon the observations presented here.

## References

[1] López-Vélez R Norman FF Bern C, 2013. 98 – American Trypanosomiasis (Chagas disease). Magill AJ Hill DR Solomon T Ryan ET, eds. Hunter’s Tropical Medicine and Emerging Infectious Disease. London, United Kingdom: W.B. Saunders, 725–738.

[2] Pan American Health Organization, 2025. *Chagas Disease*. Available at: https://www.paho.org/en/topics/chagas-disease. Accessed April 28, 2025.

[3] Bern C Kjos S Yabsley MJ Montgomery SP, 2011. *Trypanosoma cruzi* and Chagas’ disease in the United States. Clin Microbiol Rev 24: 655–681. 21976603 10.1128/CMR.00005-11 PMC3194829

[4] Bern C Messenger LA Whitman JD Maguire JH, 2019. Chagas disease in the United States: A public health approach. *Clin Microbiol Rev* 33: e00023-19. 31776135 10.1128/CMR.00023-19 PMC6927308

[5] De Lucena DT, 1957. [Persistence of living *Trypanosoma cruzi* in dead *Triatoma*]. Rev Bras Med 14: 710–711. 13527850

[6] Brener Z, 1973. Biology of *Trypanosoma cruzi*. Annu Rev Microbiol 27: 347–382. 4201691 10.1146/annurev.mi.27.100173.002023

[7] Wood SF, 1976. Influencia de la temperatura ambiental como regulador de la zoonosis por *Trypanosoma cruzi* en vectores y hospedadores. La Prensa Medica Argentina 63: 462–469.

[8] Asin SN Catala SS, 1991. Are dead *Triatoma infestans* a competent vector of *Trypanosoma cruzi*? Mem Inst Oswaldo Cruz 86: 301–305. 1842421 10.1590/s0074-02761991000300004

[9] Busselman RE Killets KC Saunders AB Hamer SA, 2025. Viable *Trypanosoma cruzi* cultured from a dead *Paratriatoma lecticularia* (Hemiptera: Reduviidae) encountered in a large dog kennel environment in south Texas, USA. J Med Entomol 62: 225–229. 39413116 10.1093/jme/tjae129

[10] Molina-Garza ZJ Mercado-Hernández R Molina-Garza DP Galaviz-Silva L, 2015. *Trypanosoma cruzi*-infected *Triatoma gerstaeckeri* (Hemiptera: Reduviidae) from Nuevo León, México, and pathogenicity of the regional strain. Biomedica 35: 372–378. 26849699 10.7705/biomedica.v35i3.2589

[11] Curtis-Robles R Auckland LD Snowden KF Hamer GL Hamer SA, 2018. Analysis of over 1500 triatomine vectors from across the US, predominantly Texas, for *Trypanosoma cruzi* infection and discrete typing units. Infect Genet Evol 58: 171–180. 29269323 10.1016/j.meegid.2017.12.016

[12] Killets KC Wormington J Zecca I Chaves LF Hamer GL Hamer SA, 2025. Comparative feeding and defecation behaviors of *Trypanosoma cruzi*-infected and uninfected triatomines (Hemiptera: Reduviidae) from the Americas. Insects 16: 188. 40003818 10.3390/insects16020188 PMC11856564

[13] Duffy T, , 2013. Analytical performance of a multiplex Real-Time PCR assay using TaqMan probes for quantification of *Trypanosoma cruzi* satellite DNA in blood samples. PLoS Negl Trop Dis 7: e2000. 23350002 10.1371/journal.pntd.0002000 PMC3547845

[14] Sadigursky M Brodskyn CI, 1986. A new liquid medium without blood and serum for culture of hemoflagellates. Am J Trop Med Hyg 35: 942–944. 3532849 10.4269/ajtmh.1986.35.942

[15] Fernandes JF Castellani O, 1966. Growth characteristics and chemical composition of *Trypanosoma cruzi*. Exp Parasitol 18: 195–202.

[16] Faraway JJ, 2014. Linear Models with R. New York, NY: Chapman and Hall/CRC.

[17] Burnham KP Anderson DR, 2004. Multimodel inference: Understanding AIC and BIC in model selection. Soc Methods Res 33: 261–304.

[18] R Core Team, 2025. R: A Language and Environment for Statistical Computing. Vienna, Austria: R Foundation for Statistical Computing.

[19] Chagas C, 1909. Nova tripanozomiaze humana: Estudos sobre a morfolojia e o ciclo evolutivo do *Schizotrypanum cruzi* n. gen., n. sp., ajente etiolojico de nova entidade morbida do homem. Memórias do Instituto Oswaldo Cruz 1: 159–218.

[20] Fiatsonu E Busselman RE Hamer GL Hamer SA Ndeffo-Mbah ML, 2023. Effectiveness of fluralaner treatment regimens for the control of canine Chagas disease: A mathematical modeling study. PLoS Negl Trop Dis 17: e0011084. 36693084 10.1371/journal.pntd.0011084 PMC9897538

[21] Rokhsar JL Raynor B Sheen J Goldstein ND Levy MZ Castillo-Neyra R, 2023. Modeling the impact of xenointoxication in dogs to halt *Trypanosoma cruzi* transmission. PLoS Comput Biol 19: e1011115. 37155680 10.1371/journal.pcbi.1011115 PMC10194993

[22] Roellig DM Ellis AE Yabsley MJ, 2009. Oral transmission of *Trypanosoma cruzi* with opposing evidence for the theory of carnivory. *J Parasitol* 95: 360–364. 18763853 10.1645/GE-1740.1 PMC2911628

[23] Maciel BJ Reigada C Digirolamo FA Rengifo M Pereira CA Miranda MR Sayé M, 2025. The potential of the antifungal nystatin to be repurposed to fight the protozoan *Trypanosoma cruzi*. *Front Microbiol* 16: 1539629. 40143876 10.3389/fmicb.2025.1539629 PMC11937040

